# Supplementary figures and images for: Bacillus subtilis encodes a discrete flap endonuclease that cleaves RNA-DNA hybrids
Source: PLoS Genet. 2023 May 5;19(5):e1010585. doi: 10.1371/journal.pgen.1010585 (PMC10191290; doi:10.1371/journal.pgen.1010585)

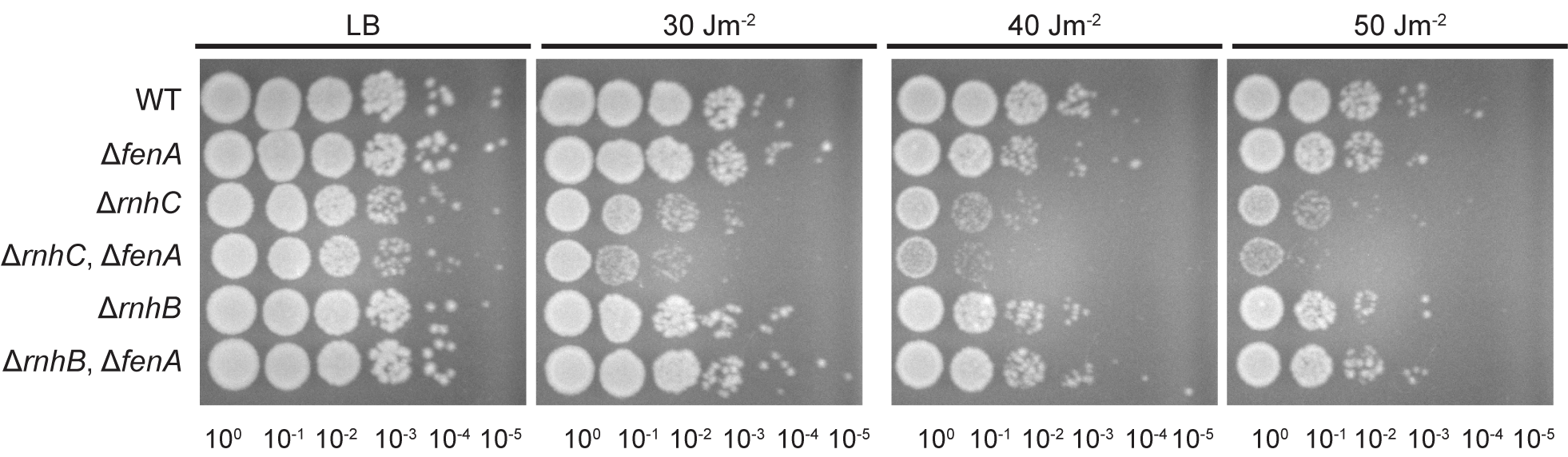

Supplement: S1 Fig — Spot titer assay of the indicated strains exposed to UV damage. (TIF) [file pgen.1010585.s001.tif]

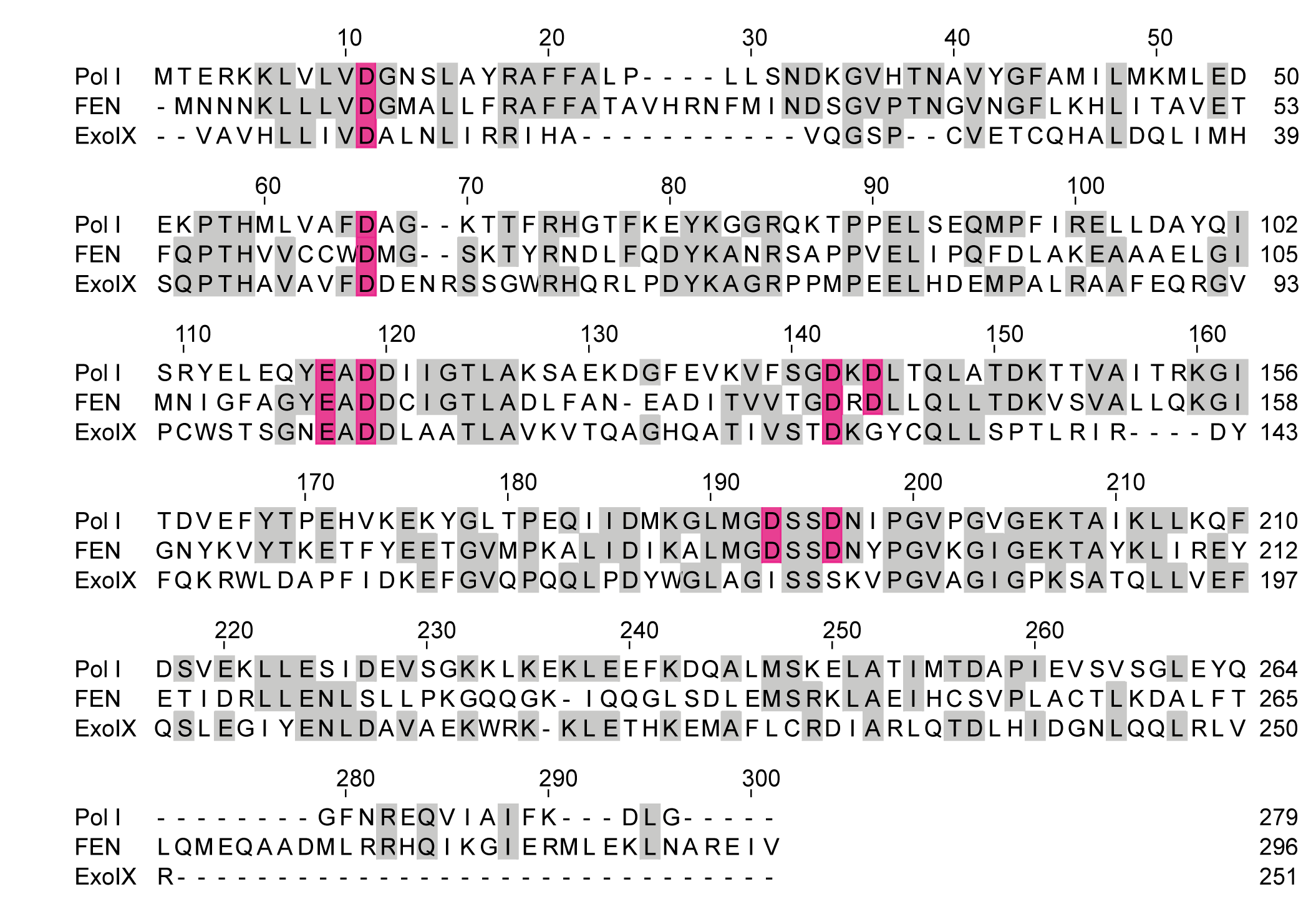

Supplement: S2 Fig — Multiple sequence alignment of Pol I (N-terminal FEN domain) and FEN from B. subtilis as well as the FEN homolog from E. coli, ExoIX. Conserved residues are boxed in grey while the active site carboxylate residues that coordinate metal-binding are boxed in pink. (TIF) [file pgen.1010585.s002.tif]

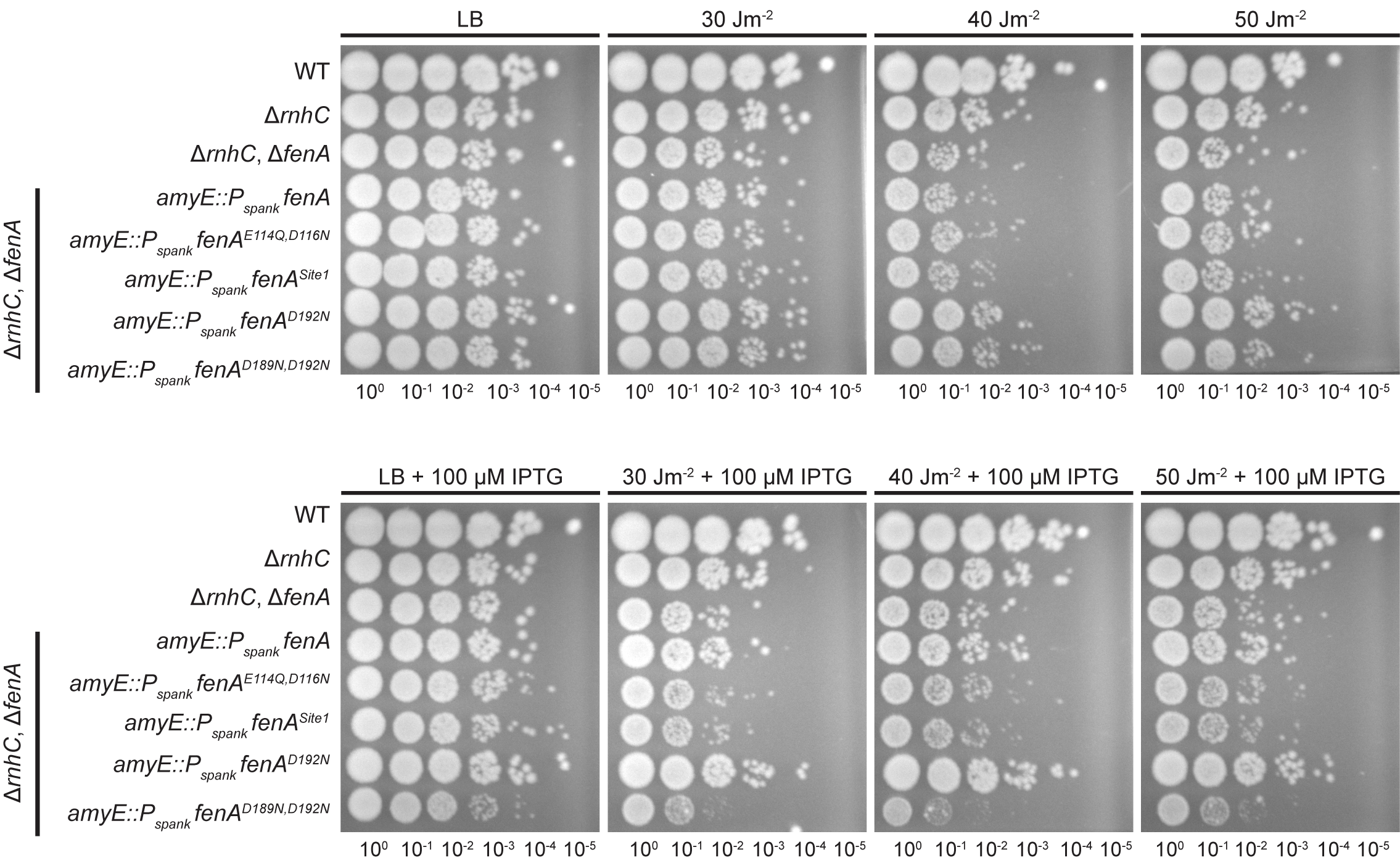

Supplement: S3 Fig — Ectopic expression of fenA and fenA mutants in the ΔrnhC, ΔfenA strain grown following UV exposure. (TIF) [file pgen.1010585.s003.tif]

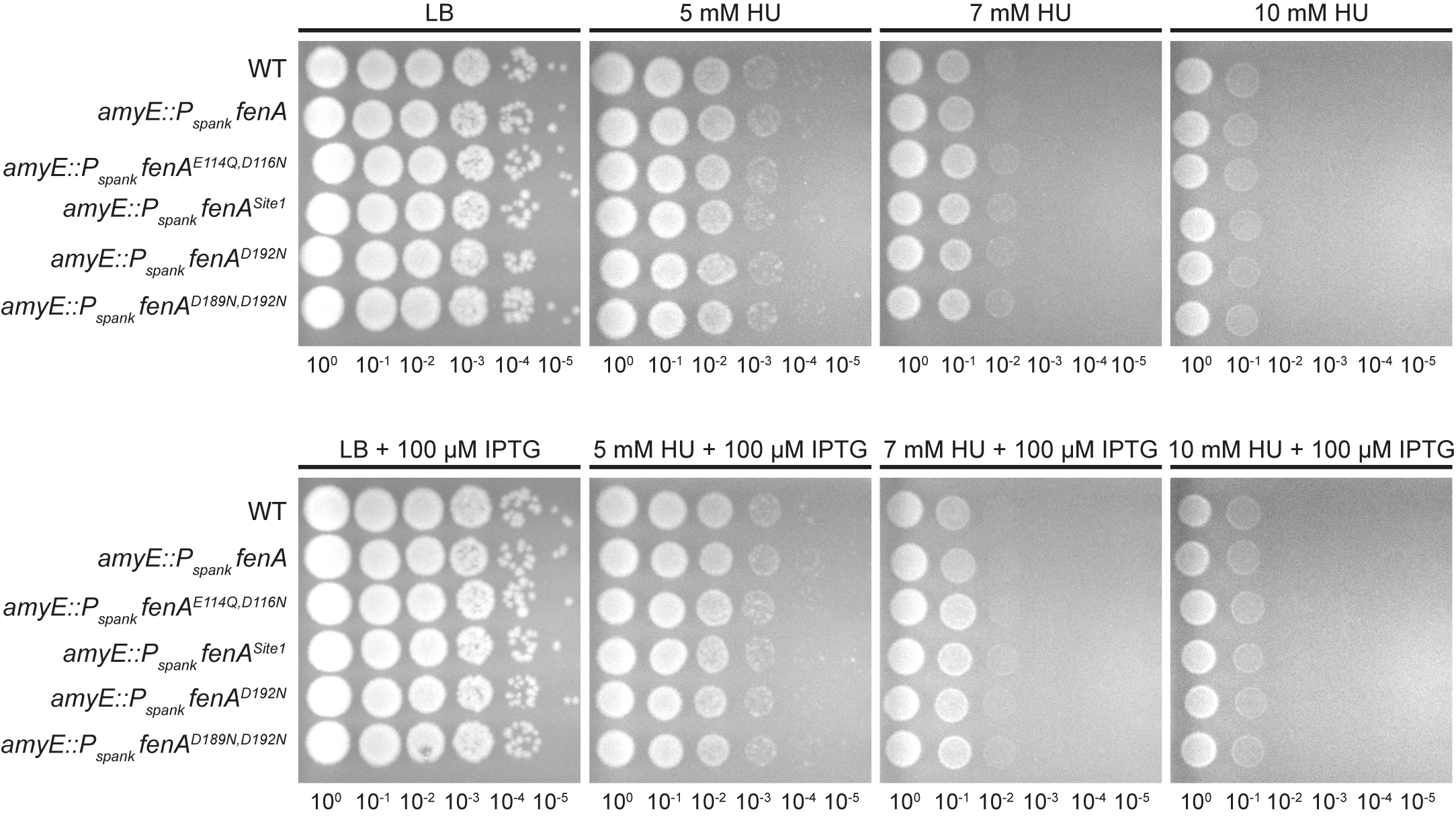

Supplement: S4 Fig — WT B. subtilis ectopically expressing fenA or fenA mutants with the indicated changes. Cells were imaged after growth at 30°C for 16 hours on the indicated concentration of hydroxyurea (HU). (TIF) [file pgen.1010585.s004.tif]

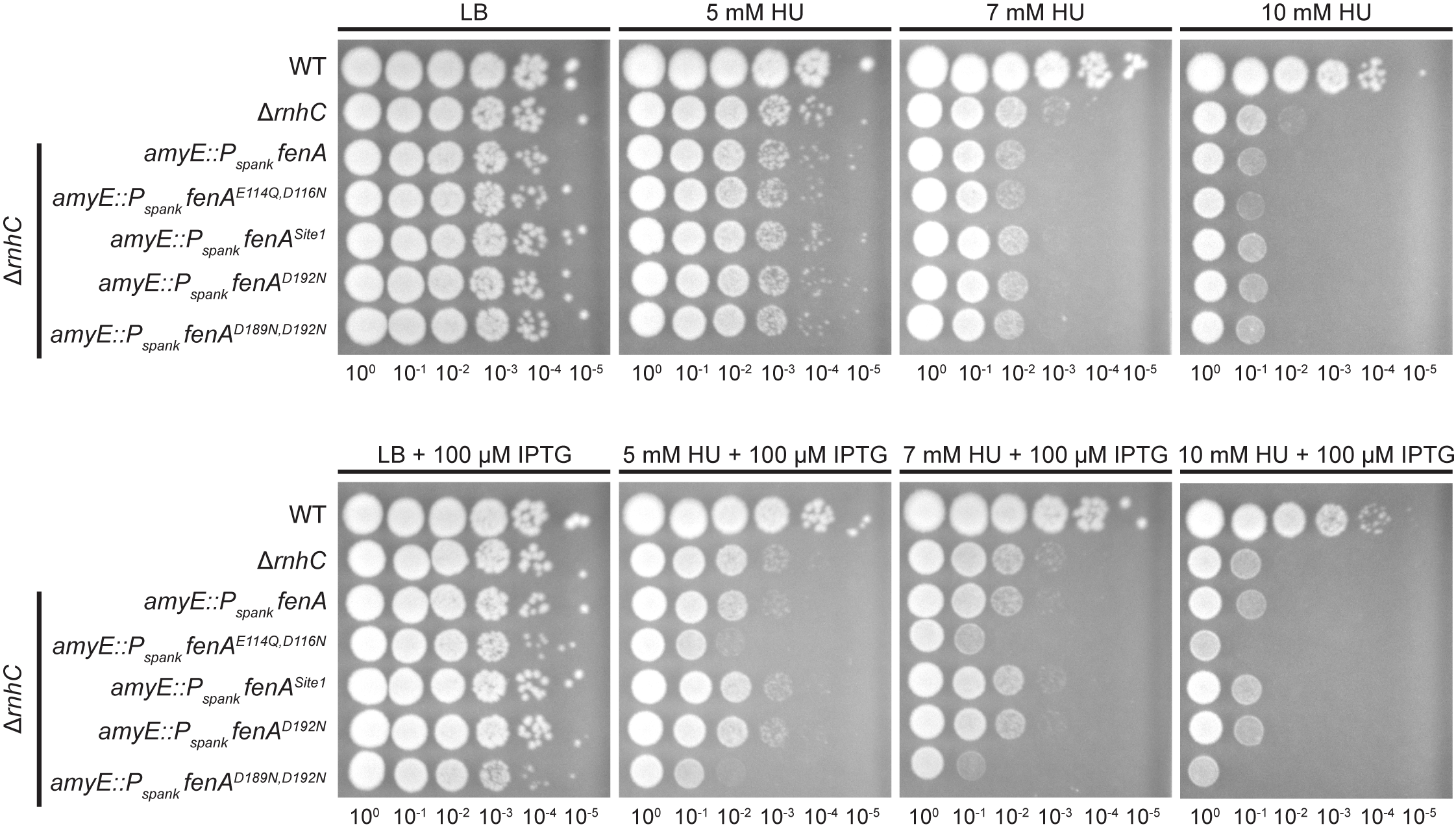

Supplement: S5 Fig — Overexpression of ectopic fenA or fenA mutants in cells lacking rnhC grown at different concentrations of hydroxyurea. (TIF) [file pgen.1010585.s005.tif]

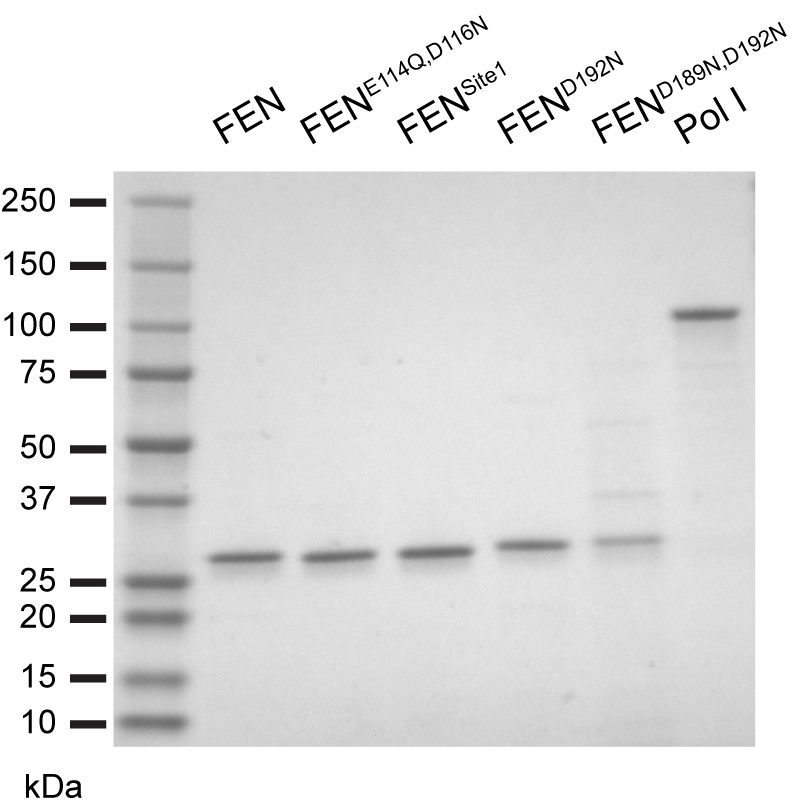

Supplement: S6 Fig — A total of 1 μg of each protein for in vitro assays purified as described in the Methods section were electrophoresed on an SDS-PAGE. The gel was visualized following staining with Coomassie brilliant blue. (TIF) [file pgen.1010585.s006.tif]

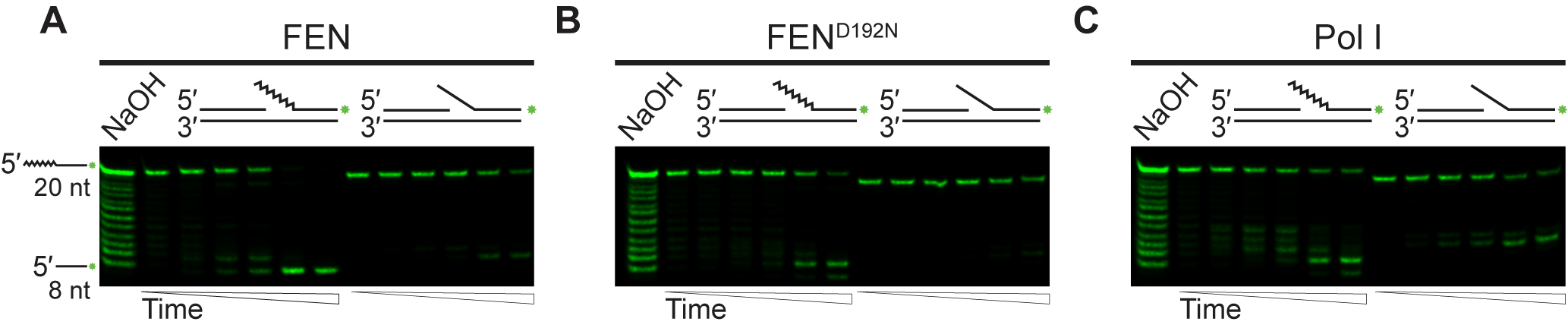

Supplement: S7 Fig — (A) Nuclease assays of FEN (B) FEND192N, and (C) Pol I on the simple flap substrate using a reaction buffer with 10 mM potassium (KCl). Substrates were generated using oligonucleotides oJR365 and either oJR339 (RNA-DNA hybrid; RNA indicated by zigzags) or oJR348 (DNA only), with a ladder produced via alkaline hydrolysis of the hybrid structure. Timepoints are as follows: 0 s, 10 s, 30 s, 1 min, 5 min, 15 min. (TIF) [file pgen.1010585.s007.tif]

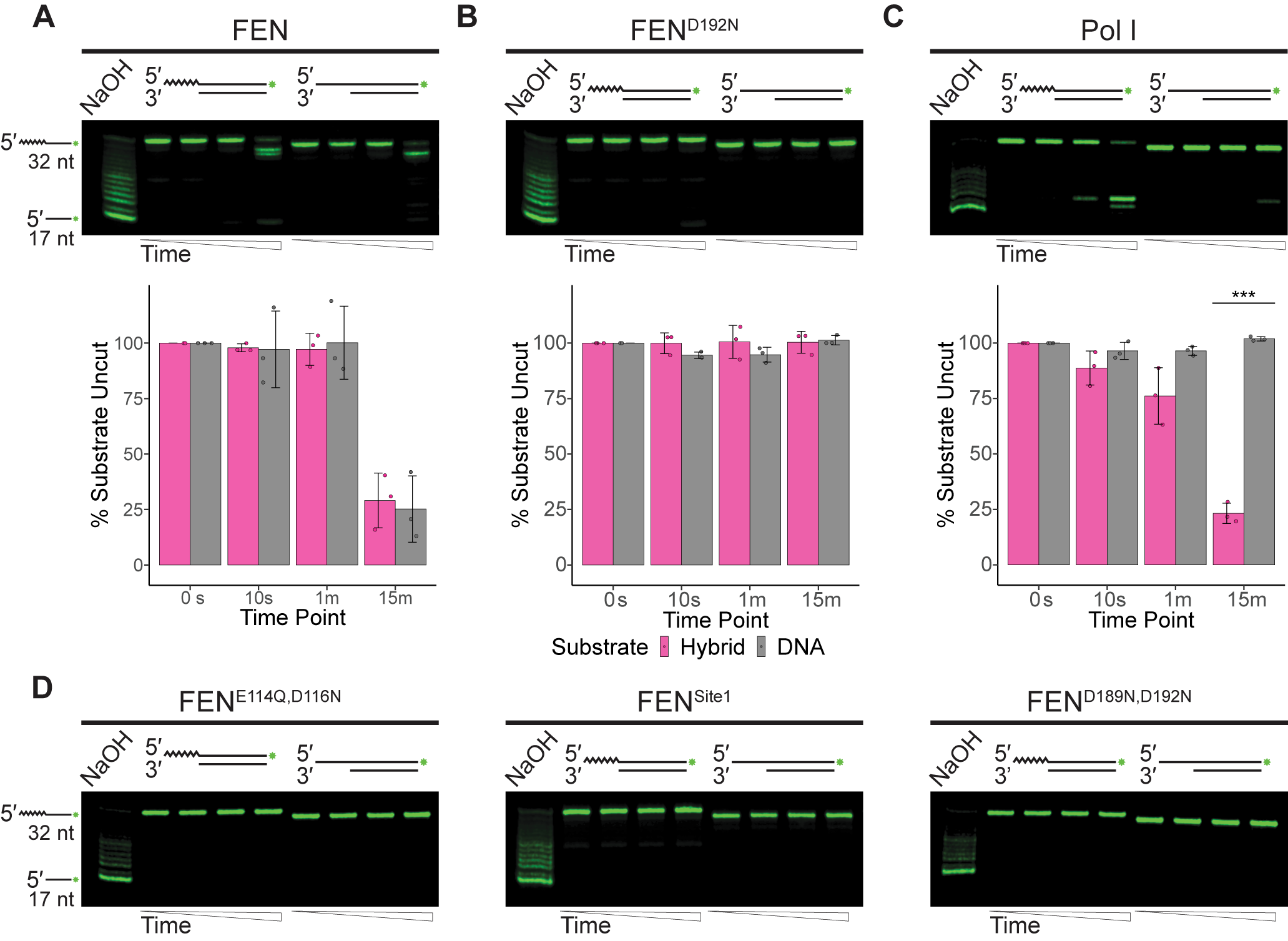

Supplement: S8 Fig — (A) Nuclease activity assays of FEN, (B) FEND192N, or (C) Pol I on duplex DNA with a 5′ overhang resolved using urea-PAGE. The mean percent substrate left intact by each protein was quantified from three replicates, shown underneath the respective assay. Standard deviation bars are provided, and statistical significance is indicated by asterisks as follows: * p<0.05, ** p<0.01, or *** p<0.001. (D) The FEN mutants FENE1414Q,D116N, FENSite1, and FEND189N,D192N were also assayed for activity on the 5′ overhang structures. 5′ overhang structures were generated by annealing oJR365 with oFCL5 (RNA-DNA hybrid; RNA indicated by zigzags) or oFCL4 (DNA). Reaction time points are 0 s, 10 s, 1 min, and 15 min. Ladder was generated via alkaline hydrolysis of the hybrid 5′ overhang structure. (TIF) [file pgen.1010585.s008.tif]

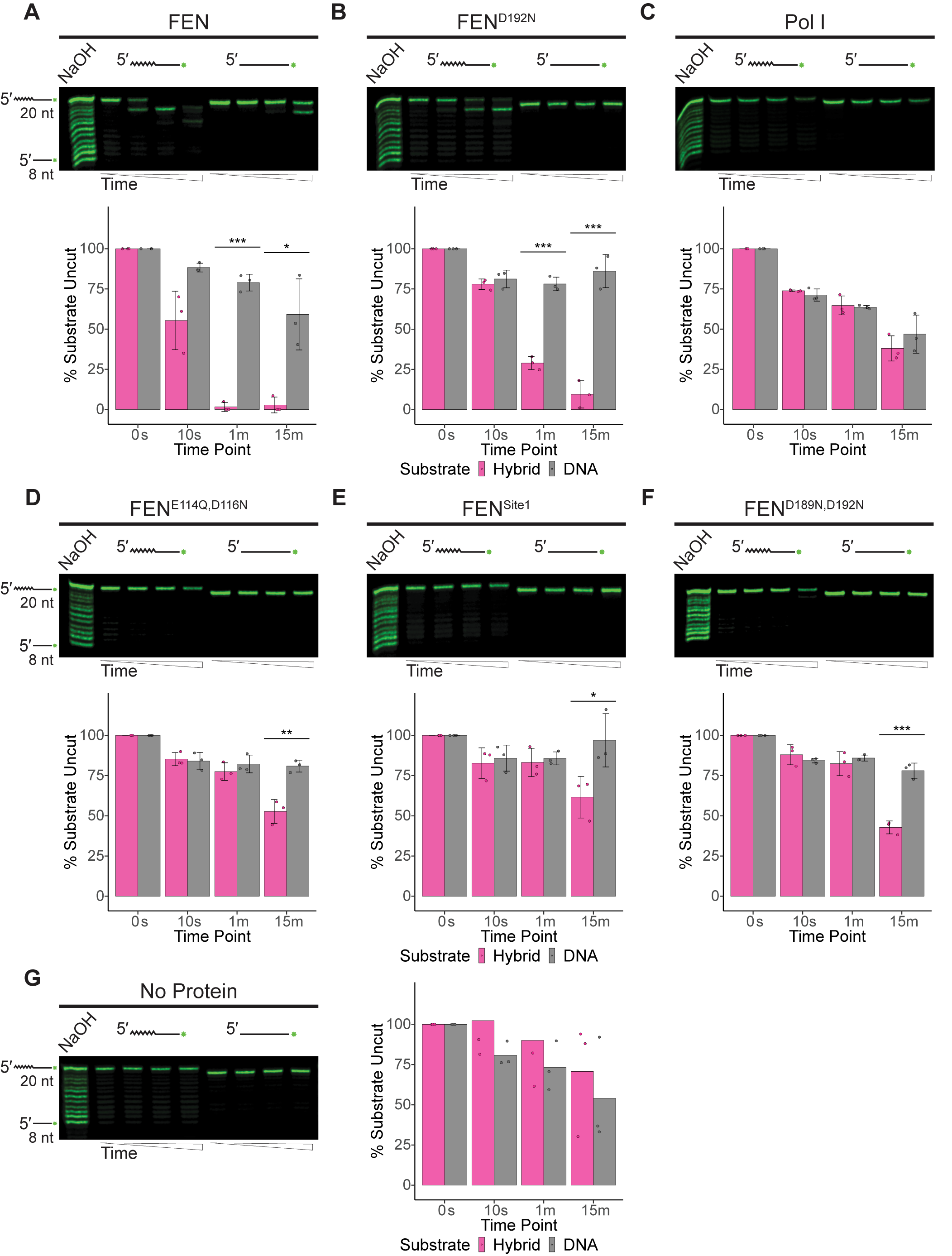

Supplement: S9 Fig — (A) Activity of FEN, (B) FEND192N, or (C) Pol I on single-stranded RNA-DNA hybrid or DNA. The mean percent of intact substrate was quantified from three replicates and shown below the appropriate gel. Significance is indicated by asterisks (* p<0.05, ** p<0.01, or *** p<0.001) and standard deviation are provided. (D-F) FEN mutants were assayed on the same substrate, with representative gels shown from at least three replicates. Quantification of three replicates is shown below the associated gel. (G) Activity assay was repeated without the addition of protein (left) and percent substrate remaining intact was visualized graphically (right). Single-stranded RNA-DNA hybrid (RNA indicated by zigzags) was oligonucleotide oJR339 while single-stranded DNA was oJR348. (TIF) [file pgen.1010585.s009.tif]
