# Supplementary material for: Bacillus subtilis encodes a discrete flap endonuclease that cleaves RNA-DNA hybrids
Source: PLoS Genet. 2023 May 5;19(5):e1010585. doi: 10.1371/journal.pgen.1010585 (PMC10191290; doi:10.1371/journal.pgen.1010585)
Supplement: S1 Table — (DOCX) [file pgen.1010585.s010.docx]

**S1_Table.doc Oligonucleotides used in *in vitro* assays.**

| **Name** | **Sequence** |
| --- | --- |
| oJR338 | GCATCAAGCGCG |
| oJR339 | rArGrUrArGrUrGrArArCrCrATGCTTACG/3IR800CWN/ |
| oJR340 | CGTAAGCATGGTTCACTACTCGCGCTTGATGC |
| oJR348 | AGTAGTGAACCATGCTTACG/3IR800CWN/ |
| oJR365 | CGTAAGCATGGTTCACTACT |
| oJR366 | GCATCAAGCGCGAGATCACT |
| oJR367 | rArGrUrArGrUrGrArArCrCrATGCTTACGAGTCGATTGC/3IRD700/ |
| oJR368 | CGTAAGCATGGTAGTGATCTCGCGCTTGATGC |
| oFCL4 | GTACTCTCCAAAAGTAGTGAACCATGCTTACG/3IR800CWN/ |
| oFCL5 | rGrUrArCrUrCrUrCrCrArArArArGrUAGTGAACCATGCTTACG/3IR800CWN/ |
| oFCL6 | GCAATCGACTCGTAAGCATGGTTCACTACTAGCTGCACATCGCTGCTTGATGCTCAATCG |
| oFCL8 | /5IRD800/C*G*A*TTGAGCATCAAGCAGCG |
| oFCL10 | GCATCAAGCGCGAGATCACTG |
| oFCL11 | /5IRD800/CGATTGAGCATCAAGCAGCGATGTGCAGCTAGTAGTGAACCATGCTTACGAGTCGATTGC |
| oFCL12 | GCATCAAGCGCGAGATCACTACCAG |

Red bases indicate ribonucleotides.

IRD indicates an infrared dye at the 5′ end or 3′ end as indicated, 700 or 800 indicates the wavelength of excitation, and CWN indicates a NHS ester conjugation.

* symbol indicates phosphorothioate bonds.
