# Supplementary material for: Bacillus subtilis encodes a discrete flap endonuclease that cleaves RNA-DNA hybrids
Source: PLoS Genet. 2023 May 5;19(5):e1010585. doi: 10.1371/journal.pgen.1010585 (PMC10191290; doi:10.1371/journal.pgen.1010585)
Supplement: S2 Table — (DOCX) [file pgen.1010585.s011.docx]

**S2_Table. All primers used in this study.**

| **Primer** | **Sequence** | | **Purpose** |
| --- | --- | --- | --- |
| oPEB866 | | TTTATGCAGCAATGGCAAGAAC | Sequencing pDR110 inserts (F) |
| oPEB867 | | GCCGACTCAAACATCAAATCTTAC | Sequencing pDR110 inserts (R) |
| oJR46 | | TCGAGCACCACCACCACCACCACTGAG | Amplifying pE-SUMO vector (F) |
| oJR47 | | ACCTCCAATCTGTTCGCGGTGAGCCTCAATAATATCG | Amplifying pE-SUMO vector (R) |
| oJR260 | | CGACTAAGGAGGTATACATATGACGGAACGAAAAAAATTAGTGC | Inserting *polA* into pDR110 (F) |
| oJR261 | | GCTTGCATGCGGCTAGCTTATTTCGCATCGTACCAAGATGG | Inserting *polA* into pDR110 (R) |
| oJR262 | | GCTAGCCGCATGCAAGCTAA | Amplifying pDR110 vector (F) |
| oJR263 | | ATGTATACCTCCTTAGTCGACTAAGCTTA | Amplifying pDR110 vector (R) |
| oJR344 | | TCGACTAAGGAGGTATACATATGAATAATAATAAACTATTGCTGGTTGACGGC | Inserting *fenA* into pDR110 (F) |
| oJR345 | | TTAGCTTGCATGCGGCTAGCCTAAACGATCTCTCTAGCGTTCAGC | Inserting *fenA* into pDR110 (R) |
| oJR378 | | GCAGCGGCTGCCTGTATCGGTACTCTTGCCG | Creating *fen^Site1^* mutation (F) |
| oJR379 | | CAGGCAGCCGCTGCATAGCCGGCAAAGCC | Creating *fen^Site1^* mutation (R) |
| oJR404 | | GATTCCAGTAATAACTATCCCGGAGTCAAAG | Creating *fen^D192N^* mutation (F) |
| oJR405 | | GGATAGTTATTACTGGAATCACCCATAAGCG | Creating *fen^D192N^* mutation (R) |
| prFCL25 | | CCTTTCGGGCTTTGTTAGCAGCC | Sequencing of pE-SUMO inserts |
| prFCL32 | | CAAGCGAATGACTGTATCGGTACTCTTGCCG | Creating *fen^E114Q,D116N^* mutation (F) |
| prFCL33 | | CAGTCATTCGCTTGATAGCCGGCAAAGCC | Creating *fen^E114Q,D116N^* mutation (R) |
| prFCL38 | | GTAATTCCAGTAATAACTATCCCGGAGTCAAAGG | Creating *fen^D189N,D192N^* mutation (F) |
| prFCL39 | | GATAGTTATTACTGGAATTACCCATAAGCG | Creating *fen^D189N,D192N^* mutation (R) |
| prFCL61 | | /5Phos/TAAGCTAGCCGCATGCAAGCTAATTCG | Creating pFCL27 from pFCL23 (F) |
| prFCL62 | | /5Phos/GCCAAGATCTTTAAAGATGGCAATGACC | Creating pFCL27 from pFCL23 (R) |
| prFCL65 | | /5Phos/TTCAACACGCTTCTTGAACGGCTTG | Creating pFCL16 from pFCL23 (F) |
| prFCL66 | | /5Phos/CATATGTATACCTCCTTAGTCGACTAAGC | Creating pFCL16 from pFCL23 (R) |
| prFCL67 | | CGACTAAGGAGGTATACATGTGGCTGTTCATTTGCTTATTGTCG | Inserting *xni* into pDR110 (F) |
| prFCL68 | | GCTTGCATGCGGCTAGCTTACCGTACCAACCGCAATTGC | Inserting *xni* into pDR110 (R) |
