# Supplementary material for: Bacillus subtilis encodes a discrete flap endonuclease that cleaves RNA-DNA hybrids
Source: PLoS Genet. 2023 May 5;19(5):e1010585. doi: 10.1371/journal.pgen.1010585 (PMC10191290; doi:10.1371/journal.pgen.1010585)
Supplement: S3 Table — (DOCX) [file pgen.1010585.s012.docx]

**S3_Table. All strains used in this study.**

| **Strain** | **Relevant Genotype** | **Citation** |
| --- | --- | --- |
| FCL3 | Δ*polA* | JWS235 [1] |
| FCL4 | Δ*fenA* | JRR94 [1] |
| FCL5 | Δ*rnhC*, Δ*fenA* | JRR96 [1] |
| FCL10 | native PY79 | Youngman [2] |
| FCL11 | Δ*rnhC* | JRR48 [3] |
| FCL12 | Δ*rnhC*, Δ*polA* | JRR64 [1] |
| FCL13 | *E. coli* MC1061 |  |
| FCL14 | *E. coli* BL21(DE3) |  |
| FCL28 | Δ*rnhC*, Δ*fenA*, *amyE*::*P_spank_ fenA* (spec^R^) | This work |
| FCL39 | Δ*polA*, *amyE*::*P_spank_ polA_Klenow_* (spec^R^) | This work |
| FCL54 | Δ*polA*, *amyE*::*P_spank_ polA* (spec^R^) | This work |
| FCL60 | Δ*rnhC*, Δ*fenA*, *amyE*::*P_spank_ polA* (spec^R^) | This work |
| FCL62 | Δ*rnhC*, *amyE*::*P_spank_ fenA* (spec^R^) | This work |
| FCL65 | Δ*rnhC*, Δ*fenA*, *amyE*::*P_spank_ fenA^E114Q,D116N^* (spec^R^) | This work |
| FCL66 | Δ*rnhC*, Δ*fenA*, *amyE*::*P_spank_ fenA^Site1^* (spec^R^) | This work |
| FCL67 | Δ*rnhC*, Δ*fenA*, *amyE*::*P_spank_ fenA^D189N,D192N^* (spec^R^) | This work |
| FCL68 | Δ*rnhC*, Δ*fenA*, *amyE*::*P_spank_ fenA^D192N^* (spec^R^) | This work |
| FCL74 | *amyE*::*P_spank_ fenA* (spec^R^) | This work |
| FCL75 | *amyE*::*P_spank_ fenA^E114Q,D116N^* (spec^R^) | This work |
| FCL76 | *amyE*::*P_spank_ fenA^Site1^* (spec^R^) | This work |
| FCL77 | *amyE*::*P_spank_ fenA^D189N,D192N^* (spec^R^) | This work |
| FCL78 | *amyE*::*P_spank_ fenA^D192N^* (spec^R^) | This work |
| FCL81 | Δ*polA*, *amyE*::*P_spank_ polA_FEN_* (spec^R^) | This work |
| FCL86 | Δ*rnhC*, Δ*fenA*, *amyE*::*P_spank_ xni* (spec^R^) | This work |
| FCL87 | Δ*rnhC*, Δ*fenA*, *amyE*::*P_spank_ polA_fen_* (spec^R^) | This work |
| FCL103 | Δ*rnhC*, *amyE*::*P_spank_ fenA^E114Q,D116N^* (spec^R^) | This work |
| FCL104 | Δ*rnhC*, *amyE*::*P_spank_ fenA^Site1^* (spec^R^) | This work |
| FCL105 | Δ*rnhC*, *amyE*::*P_spank_ fenA^D189N,D192N^* (spec^R^) | This work |
| FCL106 | Δ*rnhC*, *amyE*::*P_spank_ fenA^D192N^* (spec^R^) | This work |
| FCL124 | Δ*rnhB*, Δ*fenA* | This work |
| FCL126 | Δ*rnhB* | JRR25 [3] |

References

1. Randall JR, Nye TM, Wozniak KJ, Simmons LA. RNase HIII is important for Okazaki fragment processing in Bacillus subtilis. J Bacteriol. 2019;201: e00686–e00699. doi:10.1128/jb.00686-18

2. Youngman P, Perkins JB, Losick R. Construction of a cloning site near one end of Tn917 into which foreign DNA may be inserted without affecting transposition in Bacillus subtilis or expression of the transposon-borne erm gene. Plasmid. 1984;12: 1–9. doi:10.1016/0147-619x(84)90061-1

3. Yao NY, Schroeder JW, Yurieva O, Simmons LA, O’Donnell ME. Cost of rNTP/dNTP pool imbalance at the replication fork. Proc National Acad Sci. 2013;110: 12942–12947. doi:10.1073/pnas.1309506110
