# Supplementary material for: Bacillus subtilis encodes a discrete flap endonuclease that cleaves RNA-DNA hybrids
Source: PLoS Genet. 2023 May 5;19(5):e1010585. doi: 10.1371/journal.pgen.1010585 (PMC10191290; doi:10.1371/journal.pgen.1010585)
Supplement: S4 Table — (DOCX) [file pgen.1010585.s013.docx]

**S4_Table. All plasmids used in this study.**

| **Plasmid** | **Vector** | **Insert** |
| --- | --- | --- |
| pJR22 | pE-SUMO | *polA* [1] |
| pJR31 | pE-SUMO | *fenA* [2] |
| pJR80 | pDR110 | *fenA* |
| pFCL12 | pE-SUMO | *fenA^Site1^* |
| pFCL15 | pE-SUMO | *fenA^E114Q,D116N^* |
| pFCL16 | pDR110 | *polA_Klenow_* |
| pFCL17 | pDR110 | *xni* |
| pFCL18 | pE-SUMO | *fenA^D189N,D192N^* |
| pFCL19 | pDR110 | *fenA^Site1^* |
| pFCL20 | pDR110 | *fenA^E114Q,D116N^* |
| pFCL21 | pDR110 | *fenA^D189N,D192N^* |
| pFCL23 | pDR110 | *polA* |
| pFCL24 | pDR110 | *fenA^D192N^* |
| pFCL27 | pDR110 | *polA_fen_* |
| pFCL28 | pE-SUMO | *fenA^D192N^* |

References

1. Schroeder JW, Randall JR, Hirst WG, O’Donnell ME, Simmons LA. Mutagenic cost of ribonucleotides in bacterial DNA. Proc National Acad Sci. 2017;114: 11733–11738. doi:10.1073/pnas.1710995114
2. Randall JR, Nye TM, Wozniak KJ, Simmons LA. RNase HIII is important for Okazaki fragment processing in Bacillus subtilis. J Bacteriol. 2019;201: e00686–e00699. doi:10.1128/jb.00686-18
