# Supplementary material for: Bacillus subtilis encodes a discrete flap endonuclease that cleaves RNA-DNA hybrids
Source: PLoS Genet. 2023 May 5;19(5):e1010585. doi: 10.1371/journal.pgen.1010585 (PMC10191290; doi:10.1371/journal.pgen.1010585)
Supplement: Attachment — Submitted filename: Lowder and Simmons PLOS Genetics review_FCL_LAS.pdf [file pgen.1010585.s015.pdf]

Reviewer #1: Summary

The authors use Bacillus subtilis as a model to investigate the role of a flap endonuclease (FEN) protein in removal of RNA primers in Okazaki fragment maturation. They use genetic and biochemical approaches to do this which mainly take the form of endonuclease assays on model oligonucleotide substrate. The authors found that the B. subtilis protein FEN is involved in Okazaki fragment maturation through growth assays done under DNA damaging conditions and observed increased sensitivity to DNA damage in the absence of both FEN and ribonuclease HIII. To understand FEN's activity in Okazaki fragment maturation, they purified the protein and measured endonuclease activity on a variety of substrates that mimic RNA-DNA hybrids that form during Okazaki fragment synthesis and those that are structurally similar but are exclusively constructed from DNA. They determined that FEN has increased activity on substrates that contain portions that are RNA compared to DNA-only substrates. They also observed increased activity on structures mimicking Okazaki fragments compared to structures not expected in this pathway.

Critique

Okazaki fragment maturation is an important biological problem and B. subtilis is a good model system in which to study this. Additionally, FENs are ubiquitous in biology, so the activities learned in this system may shed light on other DNA metabolic pathways and pathways in other organisms. The work in this manuscript is very interesting and experiments appear to be well controlled. On the whole, the text is very straightforward and easy to read. One criticism, though, is that at times in the interest of conciseness and brevity, some additional nuance and analysis of the data are omitted from being discussed. This is detailed below in addition to other points.

We appreciate the referee's positive comments on our manuscript. We have edited the manuscript in accordance with the suggestions below.

• Although an elegant model, the impact of this work suffers a bit because of the similarities between the proposed pathway and pathways already established in eukaryotes. I don't think that this undermines the importance of the work, but the authors should add more in the discussion about this. E.g.--because they are similar, and because bacterial models are easier than eukaryotic, more detailed mechanistic work could be done using the B. subtilis system that will elucidate not just bacterial pathways but also eukaryotic.

This is an important point. We have included addition text in the discussion bring out the significance of our findings in the bacterial system and how future studies of *B. subtilis* can inform on lagging strand replication in more complex systems. We have added significance to bacterial replication in lines 448-461 and to eukaryotic replication in lines 475-478.

• It's interesting that the fenA-D192A rescues the double deletion phenotype in Figure 1D and has reduced catalytic activity in to the biochemical experiments. The authors mention that previously this mutant was suggested to be catalytically inactive when clearly the authors show activity. It would be helpful to add a brief explanation for this discrepancy.

The difference between the prior paper that did not show activity and the current is that the prior paper used a lower protein concentration (4 nM) relative to 50 nM used here. The other factor is

that the 3' overhang is not the ideal substrate for FEN. Therefore, the combination of less protein along with a more difficult substrate to incise resulted in no activity being detected in the former publication. We have explained this in the text of the revised manuscript lines 212-213.

• The data in Figure 1 is clear, but it would strengthen the author's argument for a role for FEN RNA primer removal and Okazaki fragment maturation if there is also a phenotype with deletion of another protein in the pathway (can ligase be used?). This may not be possible, but an alternative may be to show a lack of phenotype with a double deletion of fenA and a RNase not predicted to play a role in Okazaki fragment processing as a control.

This is an important consideration. The problem as the reviewer indicates is that ligase is essential in *B. subtilis* so we can't delete *ligA*. To address this comment, we have repeated Figure 1 and combined Δ*fenA* with an Δ*rnhB* deletion. This double mutant does not have a phenotype, demonstrating the effect is specific for Δ*fenA* and Δ*rnhC.*

• It would be helpful in the endonuclease assays, if the ladder, gel, and substrate cartoons could be labeled with sizes of the cleavage products. It appears that FEN is cleaving at a discrete position that is inferred to be at the RNA-DNA segment junction point on the strand. It would be useful to show that.

We have added the sizes of the cleaved products to each figure as requested.

• Relatedly, it would be helpful if the authors could provide additional commentary and discussion about their observed activities of FEN. For instance, it is clearly cleaving the DNA at a specific site (there is a single product band and opposed to a ladder or smear). This suggests a specific substrate recognition ability. Additionally, quantitatively comparing cleavage rates for each substrate and protein/variant would strengthen the author's presentation of data. The authors' data is quite clear and seems to contain more mechanistic observations where additional analysis would be interesting.

We have included more commentary on the activities of FEN in relation to both RNA and DNA cleavage. We have presented our data quantitatively, however not in a way that will provide rate information. We really tried to provide rate information on the substrates but were unable to do so during the time allotted for revision of this work. We hope the referee can understand that providing rate information for two different proteins on 16 substrates is a huge undertaking for an assay using a gel-based readout. We also hope the referee can appreciate that repeating all substrates to achieve cleavage rates would take at least 6 months. While it would provide more information, as the referee points out, the data we present is already quite clear. We feel that rate information would be better suited for a follow up study where rate information is the central focus of the study.

• I am confused about the disappearance of the green labeled primer strand in lane 6 in Figure 8. Does it get extended, but to a variety of lengths, so it does not resolve as a single band?

Yes, that is correct. At that time point we don't have enough full extension to generate a clear band when the channels are overlayed. When we image the green channel only, the smear is

more prevalent and obvious. When the green and red channels are overlayed it becomes difficult to see the smear. We present an enhanced version of the green channel only so that the referee can see the smear.

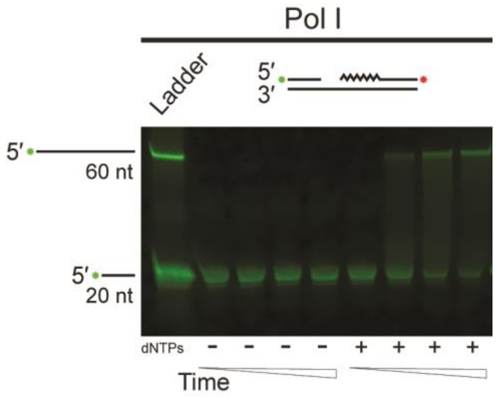

• Also, in this figure, what is the high molecular weight product in lane 7? Does Pol I extend beyond the template strand? If so, how?

This was our mistake. We used the incorrect size standard in the assay. We have repeated the assay and included the correct sizing standard.

• It would also strengthen the model, if the authors could use this assay to partially reconstitute. If they add both Pol I and FEN can they observe primer extension and RNA flap removal? They may need a nuclease deficient Pol I to do this.

We completely agree with the referee's suggestion. We have now partially reconstituted the reaction and shown that Pol I does not remove the flap on its own, but the addition of FEN allows for flap cleavage. We feel the inclusion of this new data strengthens our model.

Reviewer #2: Lowder and Simmons examine the activity and roles of B. subtilis FEN (aka YpcP or ExoR) in vitro and in vivo. Their results demonstrate a role for FEN in DNA repair in vivo when RNase HIII is absent and suggest a role for FEN in processing RNA primer flap structures in Okazaki fragment processing in vivo. A systematic biochemical study reveals robust nuclease activity for FEN on a variety of substrates, with a preference for RNA:DNA hybrids and 5' DNA flaps. Purified FEN appears to be more active as a nuclease than DNA polymerase I on the same substrates, consistent with FEN's role in Okazaki fragment processing. The manuscript is very well written and the conclusions are well supported. The findings represent a significant advancement in our understanding of how FEN participates in DNA replication, suggesting an unexpected similarity between lagging strand processing in B. subtilis and eukaryotic systems.

We thank the reviewer for their kind words supporting our work.

Concerns:
(1) There appear to be some problems with Figure 1. In Figures 1B and C, D192 is not labeled. Instead, it appears that residue 192 is mislabeled as D190. A second issue is in Figure 1D. The authors state on page 8 that overexpression of the fenA D192N mutant rescued HU sensitivity whereas the fenA D189N, D192N mutant did not. However, the results are the opposite in

Figure 1D, which makes me think that the labels are switched on the two mutant strains. The authors should confirm that these are correct.

Thank you for catching our errors. We have corrected each error and yes, the labels for FenA D189N and D129N were switched with FenA D129N

(2) Metal concentrations in enzymatic assays include 10 mM MgCl(2) and 10 uM MnCl(2), which the authors note reflect the expected physiological concentrations. The reference that supports this statement is a prior paper from the Simmons group where RNases H from B. subtilis are tested in vitro in differing divalent metal concentrations. A reference to a paper that measured free divalent metal concentrations in B. subtilis would be more appropriate.

For clarity, the physiological range of free Mg in *B. subtilis* and several other bacteria is ~1 mM rather than 10 mM and for Mn that number is in the low µM range. The numbers are based on the concentrations necessary to activate metal responsive factors. For Mn, the manganese transport regulator MntR represses expression of the manganese transporter when free Mn concentrations are sufficient. The literature reported values for the same pH range from 6.3 µM to 24 µM with most measurements around 10 µM (Kliegman et al. 2006 and Ma et al., 2012). In the case of Mg, a variety of measurements have been done in several bacteria with measurements of free Mg around 0.5-1 mM (Froschauer EM. *FEMS Microbiol. Lett*. 237:49–55 and Wakeman et al., *Journal of Bacteriology* 196(6):1206-14). We have included citations and qualified the statement.

(3) Given the complex relationship of metal-dependent nucleases on divalent metal identity, it would be nice to know if the authors have examined FEN activity in MgCl(2) or MnCl(2) alone to determine whether activity requires both. This is not essential for this paper, but a study similar to ref. 12 could be a useful contribution to the literature.

We completed this experiment in the prior referenced paper (Randall et al., JB 2012; Figure 3B PMC6416905). In that work we tested several individual metals at 1 mM and found that $MnCl_2$ gave the best activity. However, 1 mM $MnCl_2$ exceeds the relevant physiological range by 100-fold. Therefore, in that assay we also tested the 10 µM $MnCl_2$, 1 mM Mg mix used here and obtained activity to nearly the same extent as 1 mM $MnCl_2$ alone. Thus, the metal mixture provides by far the best activity for a metal(s) in the physiological range.

(4) In figure 8, why is the green product observed with Pol I and dNTPs longer than the template strand?

We made an error and electrophoresed the wrong ladder. Thank you for catching our error, we have repeated the experiment and included the correct ladder.

(5) In figure 10, it appears that the strain overexpressing E. coli xni is the most sensitive to HU treatment – why would this be? Could ExoIX be acting in a dominant-negative manner? Since ExoIX can bind to SSB in E. coli, perhaps overexpressed ExoIX is occupying binding sites on SsbA and blocking proper complex formation with other SsbA binding partners?

We agree with the review that ExoIX expression is the most sensitive and we also agree that it is probably binding the SsbA-Ct or just to DNA. In fact, we find that all our FEN variants that can bind DNA give some degree of a dominant negative result. Because the effect is somewhat minor, we did not mention the dominant negative effect in the manuscript. Prompted by the reviewer's comment we have added a statement indicating that xni expression appears dominant negative perhaps through binding to SSB in Bacillus or DNA substrates.

Reviewer #3: Substrate forms an odd structure not quite the same as the classical "double-flap (5' flap with 3' single nucleotide flap" preferred by several reported FENs e.g. yeast (https://doi.org/10.1074/jbc.M606582200); https://doi.org/10.1074/jbc.M110662200; Archaeoglobus fulgidushttps://doi.org/10.1016/S0092-8674(03)01036-5 ; human, doi: 10.1038/ncomms15855 , https://doi.org/10.1074/jbc.M115.666438; bacteria https://doi.org/10.7554/eLife.62046

All organisms from bacteria to humans and even some viruses appear to require a FEN activity for replication. These metalloproteins require divalent metals such as magnesium or manganese for activity. Many bacteria possess two distinct FEN homologues which is the case in B. subtilis and this manuscript examines a pair of FEN homologues. In contrast to human FEN1 and its eukaryotic homologues, bacterial fenA gene products have received significantly less attention than other family members, with most studies concentrating on the DNAPolI-FEN domain. FENs are generally accepted as being required for Okazaki fragment processing and the observation that many bacteria contain tow paralogues is intriguing as for example Rosenberg and coworkers were unable to identify any phenotype associated with the E. coli discrete FEN hiomologue ExoIX.

In the current manuscript, the biochemical characterisation of the fenA gene product, a discrete FEN protein is compared with that of DNA polymerase I's FEN domain on a number of substrates. It also reports on a number of genetic experiments attempting to unravel the interdependencies of RNase H, FEN and DNA Pol1. These combined comparative biochemical an genetic studies lead the authors to conclude that the fenA gene product (FEN) is mainly responsible for removing the RNA portion of maturing Okazaki fragments rather than the polymerase's own FEN-domain (located on the N-terminal 1/3rd of the protein, the C-terminal carrying the polymerase and proofreading domains, equivalent to the Klenow, or large fragment in E. coli). One of their results suggests that the DNA PolI fen domain cannot rescue a deletion of the entire PolI gene. This is in stark contrast with the situation in e.g. Strep. Pneumoniae or Haemophilus influenzae and may be quite intriguing.

The manuscript itself is mostly very well written, quite comprehensive and sets the scene for the study perfectly. The results section contains 11 clearly presented figures plus additional supplementary information showing the biochemical activity the FEN protein, various mutants designed to reduce or ablate activity by altering the active site carboxylate residues required for divalent-metal ion binding.

The conclusion that the fenA gene product has exo- and endo-nuclease activities appears reasonable as is the interpretation of the requirement for divalent metal ions. The knock-out and complementation studies, etc are also clearly described and largely well presented.

We appreciate the referee's positive comments on our manuscript. We have now edited the manuscript in accordance with the referee's suggestions below.

However, the conclusion that FEN rather than PolI-fen domain furnishes the principal Okazaki-fragment-processing flap endonuclease is, in this reviewer's opinion potentially flawed for the reason given below (Major Point 1).

MAJOR POINT

1) The main substrates used for the in vitro work described do not reflect the generally accepted structure of a FEN substrate as implied in the text (lines 199-200). A FEN substrate should consist of a so-called double flap. That is a 3' single-nucleotide "flap" or invader, adjacent to the 5'-flap on the template. The 3' flap available to base pair with the template-strand nucleotide revealed when the last ribonucleotide is removed upon 5'-flap excision. This generates a double-stranded substrate with a nick suitable for DNA ligase. However, when I drew out the structure of the substrates used (Fig2, A and elsewhere) this is not the case. The product of a 12 nucleotide (the RNA region) cleavage yields a ds-DNA with a 4 base gap between the 3' end of oJR366 and DNA-only product of cleavage of oJR339 (last 9 nucleotides ATGCTTACG), when annealed to the template oJR368. This is not a classical double-flap. Several reports on the preferred classical FEN substrate concur that they consist of the "double-flap" e.g. yeast (https://doi.org/10.1074/jbc.M606582200); https://doi.org/10.1074/jbc.M110662200; Archaeoglobus fulgidus https://doi.org/10.1016/S0092-8674(03)01036-5 ; human, doi: 10.1038/ncomms15855 , https://doi.org/10.1074/jbc.M115.666438; bacteria https://doi.org/10.7554/eLife.62046; this list is not exhaustive. The substrate used has 4 bases of complementary RNA-to template DNA (9-12), thus when cleaved to the product 12 mer produce a gapped substrate unless there has been a mistake with the sequences in the paper.

The referee makes an excellent point here. We have included the double flap substrate as described by the referee and we now indicate in the text that this substrate represents the canonical FEN substrate. As the referee will see, FEN shows far more activity on the double flap substrate than Pol I. In fact, Pol I shows very little activity on this substrate at all. These data are now included in new Figure 2. We also included a second new substrate with a double flap and a gap. Pol I has the best activity on this substrate and approaches that of FEN although the FEN reaction goes to completion much more rapidly and Pol I generates a series of intermediate products suggesting exonucleolytic cleavage. These observations further support our original conclusion that FEN is primarily responsible for Okazaki fragment processing in *B. subtilis* as FEN is more active on all the Okazaki-like substrates tested.

2) The very low level of detectable flap endonuclease activity exhibited in the B. subtilis DNA PolI holoenzyme is somewhat hard to understand. Have the conditions used been explored or optimised for PolI? It is noteworthy that the conditions used in the "nick translation" (Okazaki fragment repair) figure (8) are very different from those used in the previous nuclease characterisation figures. Why is this?

The conditions have indeed been explored and we find that Pol I FEN activity is the same under all conditions we have tried. It is in part this observation that led to our study. Under all conditions tried, FEN was more active than Pol I and under most circumstances far more active. For the Pol I extension/nick translation assays, we had to use a buffer we knew would support DNA polymerase activity. We therefore used the buffer described by the McHenry group to support replication of *B. subtilis* DNA polymerases PolC and DnaE (Sanders et al., Mol. Cell 2010 PMID: 20122408). We have also used these same conditions before for Pol I extension in prior work (Schroeder et al., PNAS 2017 PMC5676920). We then included FEN in the adjacent gels to show that FEN is active under the extension conditions used for Pol I as a control. This

once again speaks to our point that FEN is robust and Pol I shows very weak FEN activity under all examined buffer conditions on virtually all substrates tested, including the canonical FEN substrate.

We also wish to make the point that *E. coli* Pol I FEN is very active and has been assayed by several labs, yet Exo IX has either weak activity or no activity depending on the publication. With *B. subtilis*, we find that FEN is very active and Pol I FEN is not, so it would appear that bacteria either have an active Pol I FEN or an active FEN but not both.

3) Nuclease activity comparisons in Figs. 2 to 7 appear to have been carried out using buffers lacking an appreciable concentration of potassium and at very high enzyme concentrations compared with DNA (100 nanoM and 50 nanoM, respectively). Earlier work has indicated that several, but not all, FENs are greatly stimulated by the presence of a potassium ion bound in the helix-2(3)-turn helix region of FEN proteins from bacteria to man.

We initially tested the effect of potassium given the earlier work mentioned by the referee. We see no stimulation of activity for either protein when potassium is added in the reaction. We have included this experiment as **S7 Fig** in the revised text.

4) The experiments in Figs. 2-7 using high concentrations of FEN protein, were essentially complete in 1 min for the WT reaction – the product which appears to be derived by endonuclease could also have been derived by a processive EXO-nuclease action. Indeed, in Fig. 2B, the "slower" mutant shows clear evidence of bands longer than the final 12 mer product at 1 min, they seem to be 13- 15 nucleotides, with no final 12 mer until 15 min. Indeed, even in Fig 2A, there is a hint of intermediate products in 10 s and 1 min lanes. Fig 3A similarly, shows roughly equal amounts of 13 and 12 mer product in the 10s lane, some 13mer but mostly 12 mer at 1 min and all 12 mer at 15 min.

We have repeated this experiment at lower protein concentrations and we do not see a ladder indicative of exonuclease activity. There is some background due to impure oligonucleotide, which may partially explain the appearance of intermediate products. Further, these results are consistent with studies of murine FEN-1 (Harrington and Lieber, EMBO 1994, PMID: 8131753) where two discrete products resulted depending on relative Mn and Mg concentrations. As in the assays with higher protein concentrations, we observe minimal exo at the 5' end followed by FEN activity at the junction releasing the large fragment. We have included a supplemental figure **S7** highlighting this, using more timepoints to demonstrate discrete cleavage.

5) Lines 310 – expression of Pol1FEN did not reduce sensitivity… fig. 9 . I may be misinterpreting this but the figure as shown, suggest the FEN domain complemented strain (lower right hand panel) shows growth much more growth at 10E-3 and 10E-4 than the Klenow complemented ones (no colonies past 10E-2)?

This was our mistake. We mislabeled the figure and we have now corrected our unfortunate error. We thank the referee and apologize for the confusion.

Minor issues

6) Line 188-189- references materials and methods. There is no M&M section, rather just Methods.

We have made this change as requested, thank you.

7) Several uses of rpm for centrifuge speeds (e.g. line 453 and elsewhere) , better to give x g (note g should be in italic).

The cells were centrifuged at 4000 x*g*.

8) OD (line 450 and elsewhere) is a defunct term, better to use A600 or A660.

We have changed all references of OD to A600.

9) While the paper is largely thoughtfully and comprehensively referenced, the authors might wish to include comparison of their PolFEN-domain complementation with the situation in Haemophilus and Streptococcus.

We have indeed included these references and discussion of this work. This work is first referenced in the results section and discussed in the context of our work in the “Discussion” lines 448-461.

10) References contain a mix of title and sentence case.

Thank you, we have addressed this in the revised version.
