# Supplementary material for: Bacillus subtilis encodes a discrete flap endonuclease that cleaves RNA-DNA hybrids
Source: PLoS Genet. 2023 May 5;19(5):e1010585. doi: 10.1371/journal.pgen.1010585 (PMC10191290; doi:10.1371/journal.pgen.1010585)
Supplement: Attachment — Submitted filename: Lowder_Response to referees.pdf [file pgen.1010585.s016.pdf]

Below please find the referee’s comments in black text and our response in blue. We wish to thank the referees for their thoughtful suggestions.

Reviewer Comments:

Reviewer #1: The authors have done a nice job responding to reviewer comments and the paper is much clearer and improved. I have no concerns that warrant additional revision and recommend publication.

Thank you!

Reviewer #2: I am satisfied with the authors revisions and am pleased to accept the paper for publication as is.

Thank you!

Reviewer #3: The manuscript has been revised appropriately.

Thank you!

One minor comment is that in line 580 they state that they carried out assays in the presence of "10 mM KCl according to ref. 41."

The quoted reference employed 100 mM KCl in ExoIX reactions (page 8359, of ref. 41, "Reactions were carried out using 50 pM annealed flap substrate in 25 mM potassium glycinate (pH 9.3), 100 mM KCl, 1 mM DTT, 0.5 mM EDTA, with or without 10 mM MgCl2."

The authors may wish to add a caveat that any differences observed maybe due to the lower KCl concentration used.

This statement quoted by the referee from the paper in question is indeed made in the Materials and Methods section, however in the results section the authors state “*In the presence of 50 mM KCl, ExoIX showed a significant increase in affinity for the Dup1 and Dup2 duplexes with* K_d*s of 45 ± 9 nM and 190 ± 60 nM, respectively*.” Further in Figure 2A and C the authors are clearly using 50 mM KCl. Given the statements in the results and the figure it would appear the authors did not use 100 mM and instead used 50 mM.

In response to this comment, we have rephrased our statement to indicate we used a lower concentration than the prior study. We would like the referee to know that we did try 100 mM KCl in our assays and found that 10 mM and 100 mM KCl yielded the same activity. Additionally, the extensions assays (Fig 8) were carried out in a buffer containing 300 mM potassium glutamate and there was little nuclease activity from Pol I.

Minor issues:

1) Line 26- "5' ssRNA" an apostrophe is incorrectly used - this should be 5 prime (symbol).

Corrected as requested.

2) Line 81 and elsewhere. The term bivalent cation is used. Divalent seems to be more widely used in the literature.

Corrected as requested.

3) Line 85, delete "small" it is redundant as "single nucleotide" is specified.

Corrected as requested.

4) Line 271, "preference opposite that of FEN" should read " preference opposite to that shown by FEN."

Corrected as requested.

5) Line 314/5 "like what is seen here with PolI" Should read "as seen here with PolI"

Corrected as requested.

6) Line 508 and elsewhere "A600" should be A (subscript 600nm)?
We made this change, but it really depends on the journal convention.

7) Line 516 "BL21-subscript-DE3-(FCL14)" Usually BL21 with the DE3 prophage is written as BL21(DE3), not subscript? Is FCL14 a supplier or is this a variant of BL21(DE3) ? Similarly, line 599.

We have made this change. FCL14 is just the strain name designation in our collection to easily catalog our materials. It is unchanged from BL21(DE3).

8) Line 549 and elsewhere - "(20mM Tris, 500mM NaCl, 1mM DTT") - spaces between numbers and units missing should be "20 mM Tris, 50 0mM NaCl, 1 mM DTT".

We have added the spaces as requested.

9) Fig 1- Please consider adding in a panel showing a picture of the entire protein, rather than just the active site. This will provide context for readers who are unfamiliar with FEN architecture.

We have added the requested panel to the figure.

10) On page 34, legend to Sup Fig. 7, KCL is used, the "L" should be lower case- KCl.

Corrected as requested.
